# Early skin-to-skin contact or incubator for very preterm infants: study protocol for a randomized controlled trial

**DOI:** 10.1186/s13063-016-1730-5

**Published:** 2016-12-12

**Authors:** Laila Kristoffersen, Ragnhild Støen, Hilde Rygh, Margunn Sognnæs, Turid Follestad, Hilde S. Mohn, Ingrid Nissen, Håkon Bergseng

**Affiliations:** 1 Department of Pediatrics, St. Olav’s University Hospital, Trondheim, Norway; 2 Department of Laboratory Medicine, Children’s and Women’s Health, Norwegian University of Science and Technology, NTNU, Trondheim, Norway; 3 Department of Clinical Services, St. Olav’s University Hospital, Trondheim, Norway; 4 Department of Public Health and General Practice, NTNU, Trondheim, Norway; 5 Department of Circulation and Medical Imaging, Norwegian University of Science and Technology, NTNU, Trondheim, Norway; 6 Department of Anesthesia and Intensive Care Medicine, St. Olav’s University Hospital, Trondheim, Norway

**Keywords:** Very preterm infants, Early skin-to-skin, Kangaroo mother care, Neonatal intensive care unit

## Abstract

**Background:**

Skin-to-skin care immediately following delivery is a common practice for term infants and has been shown to improve cardiorespiratory stability, facilitate early bonding, and promote breastfeeding. Since 2007, the use of skin-to-skin care has been practiced for preterm infants from 32 weeks of gestation in the delivery room at St. Olav’s University Hospital. In the present study we aim to investigate whether skin-to-skin care following delivery is safe, and how it affects early and late outcomes compared to standard care for very preterm infants.

**Methods/Design:**

A randomized controlled trial (RCT) of skin-to-skin care in the delivery room for very preterm infants born at gestational age 28^0^–31^6^ weeks with birth weight >1000 grams. Infants with severe congenital malformations or need of intubation in the delivery room are excluded. A detailed checklist and a flowchart were prepared for the study, and all involved professionals (neonatologists, neonatal nurses, obstetricians, anesthesiologists, midwives) participated in medical simulation training prior to study start on February 1, 2014. A consultant in neonatology and a neonatal nurse are present at all deliveries. Infants with birth weight <1500 grams receive an intravenous line with glucose, amino acids, and caffeine citrate in the delivery room. Infants with gestational age <30 weeks are routinely put on continuous positive airway pressure (CPAP). After initial stabilization, infants are randomized to skin-to-skin care or are transferred to the nursery in an incubator. Primary outcome is cognitive development at 2 years measured with the Bayley Scales of Infant Development, Third Edition. Secondary outcomes are safety defined as hypothermia, respiratory failure, and/or cardiopulmonary resuscitation, physiological stability after birth and motor, language and cognitive development at 1 year for the child, and mental health measured with the State-Trait Anxiety Inventory (STAI) at discharge, and at 3 months and 2 years after expected date of delivery for the mothers.

**Discussion:**

The study may have important implications for the initial care for very preterm infants after delivery and increase our understanding of how early skin-to-skin care affects preterm infants and their mothers.

**Trial registration:**

ClinicalTrials, NCT02024854. Registered on 19 December 2013.

**Electronic supplementary material:**

The online version of this article (doi:10.1186/s13063-016-1730-5) contains supplementary material, which is available to authorized users.

## Background

Worldwide, 15 million infants are born preterm annually [1], and preterm birth is one of the largest direct causes of neonatal mortality and morbidity [2]. Compared to full-term infants, preterm infants are at increased risk of neurodevelopmental impairments [3–5]. This also includes lower self-esteem, social relations, and quality of life in adulthood [6]. There is growing evidence that mental and behavioral problems in children born preterm last into adulthood [7–13]. These societal and medical consequences of preterm births have led to a growing interest in developmental care models to optimize neurodevelopmental outcomes. Different approaches aiming to support infants and parents have been established in neonatal intensive care units (NICUs) in recent years, and some of these early intervention programs seem to have a positive effect on long-term function up to preschool age [5, 14, 15]. The first hours after birth represent a sensitive period for the very low birthweight infant (VLBW), and mothers who see their infant within 3 hours after birth are likely to establish a more secure attachment to the infant compared to those who do not see their infant within 3 hours [16]. Parents are increasingly acknowledged as primary caregivers for their preterm-born infant; despite the need for intensive care, most NICUs try to facilitate early parent-infant bonding. Education of parents to understand subtle signs and signals from their tiny, preterm-born infants is considered an investment in an optimal home environment for the child. From being forced to separate from their newborn infant due to restrictions of visiting hours in NICUs, parents are now generally encouraged to stay with their infant as much as possible [17, 18].

Kangaroo care (KC) [19], family-centered care (FCC) [20], Newborn Individualized Development Care and Assessment Program (NIDCAP) [21, 22], and a variety of early intervention programs emphasize the importance of establishing early parent-infant interaction to support an optimal development [23–25]. Facilitating early skin-to-skin care (SSC) is one way of supporting early parent-infant bonding and is also associated with improved physiological stability [26, 27] and decreased cortisol reactivity [28]. In addition, a recent World Health Organization (WHO) guideline on interventions to improve outcomes for preterm infants, strongly recommends early SSC as thermal care for preterm infants weighing <2000 grams [29].

Skin-to-skin care in the delivery room (DR) has been studied for preterm infants from 32 weeks of gestation [30]. However, to the best of our knowledge, SSC in the DR and the operating room (OR) has not been systematically investigated for infants born at gestational age (GA) <32 weeks. Obvious obstacles to do so would be the need for medical equipment and competent personnel, usually available only in the NICU. Breathing support, need of surfactant, intravenous access, and monitoring equipment are needed in a large proportion of infants below 32 weeks. To combine medical interventions with early SSC, a trained team and formalized guidelines are required to ensure the medical safety of the infant. Specific challenges arise with early SSC in the OR after cesarean section (C-section).

Moving treatment and medical procedures from the NICU into the DR and OR requires cooperation across specialties and departments. Pediatricians and neonatal nurses have to work outside their familiar NICU environment, while gynecologists, midwives, anesthesiologists, nurse specialists in anesthesia, and operating room nurses have to adjust their work and procedures to the presence of the preterm infant on the mother’s chest.

In the present study we aim to investigate whether SSC following delivery is safe, and how it affects early and late outcomes compared to standard care for preterm infants born at GA 28^0^–31^6^ weeks.

The purpose of this article is to present the study design and a description of the intervention, as well as provide a detailed outline of how the intervention was planned and implemented in a multidisciplinary team. Schedule of enrolment, interventions, and assessment are outlined in Table 1.

**Table 1:** Schedule of enrolment, interventions, and assessment

Activity/assessment	Staff member	Approximate time to complete	Before delivery	t_0_	t_1_	t_2_	t_3_	t_4_	t_5_	t_6_	t_7_	t_8_	t_9_	t_10_	t_11_	t_12_	t_13_	t_14_
Informed consent	Neonatal nurse/physician	15 minutes	x															
Inclusion/exclusion form	Neonatal nurse	5 minutes		x														
Randomization sealed envelopes	Neonatal nurse	1 minute			x													
Heart rate	Neonatal nurse	30 sec				x	x	x	x	x								
Respiration rate	Neonatal nurse	30 sec				x	x	x	x	x								
Oxygen saturation	Neonatal nurse	30 sec				x	x	x	x	x								
Body temperature	Neonatal nurse	30 sec				x	x	x	x			x	x					
Blood glucose	Neonatal nurse	5 minutes						x^1^	x^1^		x			x				
Blood pressure	Neonatal nurse	5 minutes							x			x	x					
Maternal anxiety (STAI)	Study coordinator	15 minutes													x	x		x
Ages & Stages Questionnaire	Study coordinator	15 minutes														x		x
General movement (GMA)	Study coordinator	5 minutes														x		
Bayley Scale of Infant and Toddler Development III	Occupational therapist	60 minutes															x	x

X^1^ = the blood glucose is measured at either 90 or 120 minutes

t_0_ = During stabilization

t_1_ = After initial stabilization

t_2_ = 15 minutes after birth

t_3_ = 30 minutes after birth

t_4_ = 60 minutes after birth

t_5_ = 120 minutes after birth

t_6_ = First 24 hour (hourly)

t_7_ = Approximately 12 hours after birth

t_8_ = 10 hours after birth

t_9_ = 18 hours after birth

t_10_ = Approximately 24 hours after birth

t_11_ = At discharge

t_12_ = 3 months corrected age

t_13_ = 1 year corrected age

t_14_ = 2 years corrected age

### Aims

#### Primary aim

To study the effect of early SSC versus standard care (SC) for very preterm infants (28^0^–31^6^ weeks) on cognitive scores at 2 years corrected age, measured with the Bayley Scales of Infant Development, Third Edition (Bayley-III).

#### Secondary aims

- To assess safety of the intervention by measuring incidence of hypothermia (temperature <36 °C) or respiratory failure during the first 2 hours of life.
- To estimate the effect of the intervention on complications to prematurity (intraventricular hemorrhage (IVH) or periventricular leukomalacia (PVL), seizures, necrotizing enterocolitis (NEC), treatment for persistent ductus arteriosus (PDA), and bronchopulmonary dysplasia (BPD)).
- To estimate the effect of the intervention on physiological stability during the first 24 hours.
- To estimate the effect of the intervention on maternal mental health at discharge from the NICU and when the child’s corrected age is 3 months and again at the corrected age of 2 years by using the State-Trait Anxiety Inventory (STAI).
- To investigate if the intervention affects the fidgety type of general movements and detailed aspects of the early motor repertoire at the corrected age of 3 months, using the General Movement Assessment (GMA) and the Assessment of Motor Repertoire – 2 to 5 months (AMR).
- To estimate the effect of the intervention on neurodevelopmental outcomes including cognition, language, and motor function at the corrected age of 1 year, and language and motor function at the corrected age of 2 years assessed with Bayley-III.
- To investigate the effect of the intervention on social and emotional competence at the corrected age of 3 months and at 2 years with the Ages & Stages Questionnaire - Social-Emotional

## Methods/Design

### Project context

The study is currently being carried out at St. Olav’s University Hospital in Trondheim. Trondheim is the third largest city in Norway, and St. Olav’s Hospital serves a population of almost 446,000 and is a tertiary care center for preterm infants <30 weeks of gestation. Approximately 50 infants with GA <32 weeks are born every year at St. Olav’s Hospital.

The NICU at St. Olav’s Hospital has 21 beds, with seven beds for intensive care, seven intermediate, and seven in a step-down unit. Altogether, there are 107 nurses (many part-time) and six consultants working in the NICU. The maternity ward has two sections with seven delivery rooms each and about 120 midwives employed.

Delivery rooms are in the same building as the NICU, but on separate floors (fifth and second floor, respectively). The operating room and the postoperative ward are in the same building and at the same floor as the NICU.

### Study design

The study is designed as an RCT with randomization to skin-to-skin care (SSC) or standard care (SC) in an incubator after delivery.

### Study population

Singleton and twin preterm infants with GA 28^0^–31^6^ and a BW >1000 grams in a stable medical condition delivered either vaginally or by C-section are candidates for inclusion. Twins are randomized to the same intervention. Infants with severe malformations not compatible with life, or requiring surgery within hours to days after birth (e.g. esophageal atresia, abdominal wall defects or neural tube defects), will be excluded.

Infants who need intubation and mechanical ventilation, or CPAP with more than 40% oxygen to maintain a saturation above 90% at 10 minutes of age, are excluded. Mothers have to be awake (not under general anesthesia) during C-section.

### Intervention

For all infants of eligible women, cord milking is advised immediately following delivery and before the infant is placed on the resuscitation unit. Predefined blood samples are taken from the umbilical cord. Blood gas is taken from the umbilical artery before cord clamping, while blood for hemoglobin, leukocytes, thrombocytes, C-reactive protein (CRP) and blood typing is sampled after the cord clamping.

**Fig. 1:**
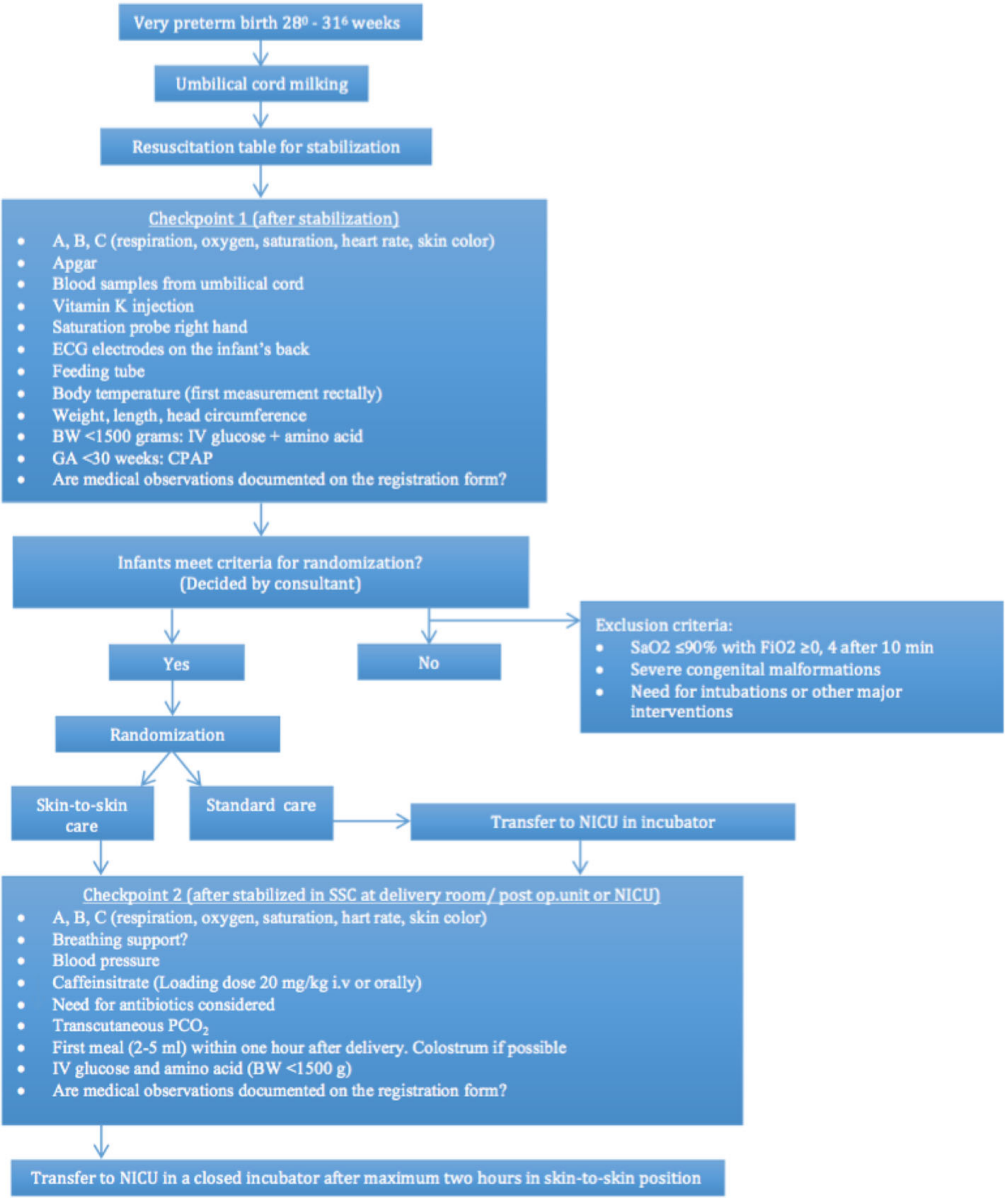
Flowchart illustrating the first 2 hours after birth

After the initial stabilization, the infant is treated and evaluated according to checkpoint 1 on the checklist, and eligibility is decided by the consultant (Fig. 1). Infants randomized to standard care are transferred to the NICU in an incubator after stabilization. The father will usually follow the newborn to the NICU, while the mother will come as soon as possible after delivery.

All infants with a BW <1500 g, irrespective of randomization, are given parenteral nutrition (glucose and amino acids) after the initial stabilization. Infants with a GA <30 weeks receive CPAP, infants ≥30 weeks get CPAP if clinically indicated. If necessary, surfactant can be administered via a thin catheter in the trachea during spontaneous breathing on CPAP (“surfactant without intubation” (SWI) if oxygen requirement exceeds 35% on CPAP and/or the infant has signs of moderate to severe respiratory distress. After the infant is stabilized in skin-to-skin position on the mother’s chest (SSC) or in a closed incubator and transferred to the NICU (SC), the infant is evaluated again (checkpoint 2) according to the checklist (Fig. 1).

Infants in the intervention group are offered SSC up to 2 hours after delivery – in the delivery room after vaginal delivery, or in the recovery room after C-section. A consultant and a neonatal nurse are responsible for the infant, while a midwife takes care of the mother. After C-section, the same personnel from the NICU and from the maternity ward are present in addition to specialist nurses from the postoperative ward. When the infant is stabilized on the mother’s chest, the consultant can leave the DR or postoperative ward in agreement with the neonatal nurse in charge. The consultant is nearby and easily reachable by telephone.

Further treatment during the hospitalization does not differ between groups. According to the unit’s guidelines, both mothers and fathers have unrestricted access to the NICU and are encouraged to have as much skin-to-skin contact with their preterm infant as possible.

### Simulation-based training (SBT)

Simulation-based training (SBT) is effective for medical education [31], facilitates multidisciplinary training [32], and can be tailored to individual needs with no risk for human patients. Before study start, simulation-based training was facilitated for five consultants, fifteen neonatal nurses and ten midwives. One gynecologist, one anesthesiologist, and one operating nurse were observers during one SBT. The SBT was a collaboration with the Medical Simulation Center (MSC) at St. Olav’s University Hospital/NTNU. The instructors from the MSC (one pediatrician and one specialist nurse) had formal European standard instructor training. An in situ scenario, reflecting the complexity of the admittance of a very preterm infant, was customized and conducted in the OR and DR during a total of seven training days.

Prior to the SBT at St. Olav’s Hospital, the teams were informed about the equipment, the environment, and the procedures for the initial stabilization and the intervention. Each trainee participated actively in one or two scenarios per training day. Immediately before the scenario, each team received a case history with information about GA and birth weight, saturation level, heart rate, and the work of breathing. Any change in physiological parameters was visible on a monitor, and the team had to act based on the infant’s condition. Each team comprised two neonatal nurses, a consultant in neonatology, and one or two midwives. One person outside the resource team acted as a mother in labor during all training days.

### Outcomes measures

#### Primary outcome

##### Bayley Scales of Infant and Toddler Development, Third Edition (Bayley-III)

The Bayley-III is a screening test that includes assessment of cognition, language (receptive and expressive), and motor function (gross and fine) in infants and young children from 0 to 42 months and provides a measurable and validated cognitive quotient [33]. The occupational therapist conducting the Bayley for ages 1 and 2 years are unaware of the intervention group.

#### Secondary outcomes

##### Safety

Hypothermia (<36 °C), respiratory failure requiring acute intubation, and/or cardiopulmonary resuscitation (CPR).

##### Physiological stability

Physiological variables are recorded during the first 24 hours (Table 1).

##### State-Trait Anxiety Inventory (STAI)

Maternal anxiety is measured with STAI Form Y [34]. STAI measures state and trait anxiety [35]; the mothers answer this questionnaire before the infants are discharged from the hospital, and when the child is 3 months and 2 years (corrected age). The questionnaire consists of 20 statements that evaluate how the mother feels ‘at this moment’ and twenty statements that evaluate how the mother feels ‘generally’. All items are scored according to a 4-point Likert scale

##### General Movement Assessment (GMA)

All infants are videotaped at 10–15 weeks post term age (fidgety movements’ period) for the GMA and the AMR. The video recordings are performed and classified according to the Prechtl method [36, 37] by a certified GMA observer unaware of the intervention group.

##### Ages & Stages Questionnaire - Social-Emotional (ASQ-SE)

The child’s social and emotional competence is evaluated using the ASQ-SE at corrected age of 3 months and 2 years [38]. The ASQ-SE comprises 22 questions that address seven behavioral areas: self regulation, compliance, communication, adaptive functioning, autonomy, affect, and interaction with people. The ASQ-SE is a screening instrument developed for children aged 3 months to 5 years.

#### Clinical registration

A case report form (CRF) has been prepared in cooperation with the Unit for Applied Clinical Research at the Norwegian University of Science and Technology (NTNU). The CRF is divided into three parts.

1. On admission and the first 24 hours:
  Background data which are recorded are: mode of delivery, GA, sex, Apgar scores at 1, 5 and 10 minutes, birthweight, length, head circumference, maternal cause of preterm delivery (preeclampsia, breech position, rupture of membranes, premature contractions or infection), fetal cause of preterm delivery (growth retardation, non-reassuring CTG registration), and antenatal steroids (full or incomplete course).
  Observations and interventions which are recorded during the first 24 hours are: oxygen saturation and requirement, mode of breathing support (hourly), surfactant administration, time of SSC after birth and any cause for interrupted SSC before 120 minutes, age at the first feed (gavage or oral), any intravenous infusion, transcutaneous carbon dioxide and total amount of enteral and parenteral nutrition given. Blood pressure is measured once during the first 120 minutes.
  Adverse events: the safety is closely monitored by the consultant and the neonatal nurse present. It will be registered as an adverse event if body temperature drops <36.0 C° or if the infants have signs of any respiratory failure requiring interventions in addition to an interruption of SSC and transfer to the NICU for a higher level of monitoring and/or respiratory support.
2. During hospitalization:
  Variables recorded are: daily weight, age on removal of feeding tube, time in skin-to-skin position every day, all nutrition (parenteral and enteral), any insulin given, any surgery, mechanical ventilation (mode and duration), CPAP/bilevel positive airway pressure (BiPAP) (duration), supplemental oxygen and/or ventilator support at 28 postnatal days, 36 and 40 weeks postmenstrual age (BPD), any sepsis (with maximal CRP and duration of antibiotic), cerebral ultrasound and magnetic resonance imaging (MRI) results (IVH or PVL), seizures, NEC, treatment for PDA and postmenstrual age (PMA) for transfer from intermediate to the step-down unit in the NICU.
3. At discharge:
  Variables recorded are: PMA, weight, length, head circumference and mode and type of nutrition. At St. Olav’s Hospital, families living less than 30 minutes by car from the hospital are offered home care with gavage feeding for infants from PMA 34 weeks. Criteria for participation for the infants are: full enteral feeds; no apneas/bradycardias requiring caffeine or monitoring; stable body temperature and positive weight gain. A family is invited to participate in the home care program if there are two caregivers at home during the day and at least one of them speaks Norwegian or English.

#### Sociodemographic information

Information about education, employment (part- or full-time) and marital status is collected from the mothers’ medical records.

#### Consent and enrolment

Pregnant women admitted to the maternity ward at St. Olav’s University Hospital for anticipated preterm delivery between 28^0^–31^6^ weeks of gestation are eligible. Oral and written information about the study is provided by a pediatrician and/or a neonatal nurse, and written consent is obtained before delivery,

#### Randomization and allocation concealment

The randomization is done after the initial stabilization of the infant (Fig. 1). Infants are stratified by weeks of gestation (28^0^–29^6^ and 30^0^–31^6^). The randomization is conducted using sealed envelopes organized by the Unit for Applied Clinical Research at the NTNU.

#### Sample size and statistical analyses

Primary analysis: sample size calculations are performed for a two-sample *t* test for comparing the Bayley Scale of Infant and Toddler Development, Third Edition, cognitive scale between the intervention (SSC) and control (SC) groups at 2 years of corrected age. To obtain a power of 80% for detecting a difference of 7.5 in mean score, using SD = 15 and significance level α = 0.05, 64 preterm infants are needed in each group. To allow for withdrawals, the sample size is set to 68 in each group.

Secondary analysis: differences between the SSC and SC groups for continuous variables will be analyzed using two-sample *t* tests, or Mann-Whitney *U* tests for data with non-normal distributions. Categorical variables will be analyzed by the Pearson chi-square test or Fisher’s exact test. *P* values <0.05 are considered statistically significant. Due consideration of multiple testing will be made when interpreting the results. All data will be presented and analyzed in accordance with the updated CONSORT guidelines for randomized trials [39].

## Discussion

The presentation of the study protocol covering design, outcome measures, sample size calculations, and procedures of this RCT on early skin-to-skin for very preterm infants, is in accordance with the Standard Protocol Items: Recommendations For Interventional Trials (SPIRIT) 2013 statement for clinical trial protocols Additional file 1 [40].

Bayley Scales of Infant and Toddler Development, Third Edition (Bayley-III) at 2 years of corrected age is chosen as the primary outcome measure. Although many interventions in the neonatal period appear to have short-term beneficial effects [15, 23, 41, 42], such effects seem to disappear over time [15, 43]. The complexity of neurodevelopment and the genetic, epigenetic, and environmental factors, which may influence the development, makes it less likely that a single, short-lasting intervention will lead to a change in cognition and/or behavior after 2 years. Thus, any intervention offered to these infants must be followed over time to learn more about their potential effects or lack of such.

In order to assess developmental delay in the children at the corrected age of 3 months and 2 years, the ASQ-SE is used [38]. This screening instrument is found to be valid in identifying of social and emotional difficulties in children [44]. Preterm delivery is associated with maternal anxiety and stress [45–47], and the effect of separation of the mother and her very preterm infant is not well studied. We have chosen to assess this aspect by using the STAI, which is validated in a Norwegian population and found reliable in terms of assessing anxiety in women [34].

Lately, there has been a shift toward a more gentle initial handling of preterm neonates. This view is well described by Jobe and colleagues, who argue that the majority of preterm infants need only supportive treatment in the transition to the extrauterine life, and not resuscitation [48, 49]. The main focus of this approach has, so far, been on early CPAP [50, 51], less invasive ways of surfactant administration [52, 53], avoidance of early mechanical ventilation [54], and maintenance of adequate body temperature [55]. In line with this, facilitation of early skin-to-skin contact between the preterm infant and the mother could contribute to a more gentle adjustment to extrauterine life with improved physiological stability. The results from this study will have important implications for how we care for very preterm infants immediately after delivery. It will also increase our understanding of how early SSC affects very preterm infants and their mothers.

Those who are conducting the Bayley-III and the GMA will be masked to the intervention group, but not the parents and NICU staff caring for the baby in the first 24 hours. Parents in the SSG group may continue to have an increased focus on SSC, and this could potentially extend the total skin-to-skin time in the NICU. However, this limitation seems inevitable with the intervention in question.

### Trial status

The study is currently recruiting participants. Since study start February 1, 2014 and through June 2016, 67 eligible pregnant women have been admitted to the maternity ward at St. Olav’s Hospital. A majority of these did not deliver before 32 weeks of gestation. Up to June, 2016, 27 infants have been included in the study.

From January 1, 2017, two other hospitals in Norway will participate in the study: Drammen Hospital, located in the eastern part of Norway, and Kristiansand Hospital in the southern part. Between 30 and 35 infants with GA <32 weeks are born every year at each of these hospitals. Based on this, the estimated time for end of inclusion will be 2018.

## Additional file

Additional file 1: SPIRIT 2013 Checklist: recommended items to address in a clinical trial protocol and related documents*. (PDF 74 kb)

## Abbreviations

AMR: Assessment of Motor Repertoire
ASQ-SE: Ages & Stages Questionnaire - Social-Emotional
Bayley-III: Bayley Scales of Infant and Toddler Development, Third Edition
BPD: Bronchopulmonary dysplasia
CPAP: Continuous positive airway pressure
CRF: Case report form
CRP: C-reactive protein
DR: Delivery room
FFC: Family-centered care
GA: Gestational age
GMA: General Movement Assessment
IVH: Intraventricular hemorrhage
KC: Kangaroo care
NEC: Necrotizing enterocolitis
NICU: Neonatal intensive care unit
NIDCAP: Newborn Individualized Developmental Assessment Program
OR: Operation room
PDA: Persistent ductus arteriosus
PMA: Postmenstrual age
PVL: Periventricular leukomalacia
SBT: Simulation-based training
SC: Standard care
SSC: Skin-to-skin care
STAI: State-Trait Anxiety Inventory
